# Nuclear proteome of virus-infected and healthy potato leaves

**DOI:** 10.1186/s12870-020-02561-7

**Published:** 2020-07-29

**Authors:** Minna-Liisa Rajamäki, Sidona Sikorskaite-Gudziuniene, Nandita Sarmah, Markku Varjosalo, Jari P. T. Valkonen

**Affiliations:** 1 Department of Agricultural Sciences, University of Helsinki, PO Box 27, FI-00014 Helsinki, Finland; 2 Institute of Horticulture, Lithuanian Research Centre for Agriculture and Forestry, Kaunas Street 30, Babtai, LT-54333 Kaunas District, Lithuania; 3 Institute of Biotechnology, University of Helsinki, PO Box 56, FI-00014 Helsinki, Finland

**Keywords:** Potato virus A, Potyvirus, Potato, *Solanum*, Nucleus, Proteome

## Abstract

**Background:**

Infection of plants by viruses interferes with expression and subcellular localization of plant proteins. Potyviruses comprise the largest and most economically damaging group of plant-infecting RNA viruses. In virus-infected cells, at least two potyviral proteins localize to nucleus but reasons remain partly unknown.

**Results:**

In this study, we examined changes in the nuclear proteome of leaf cells from a diploid potato line (*Solanum tuberosum* L.) after infection with potato virus A (PVA; genus *Potyvirus*; Potyviridae) and compared the data with that acquired for healthy leaves. Gel-free liquid chromatography–coupled to tandem mass spectrometry was used to identify 807 nuclear proteins in the potato line v2–108; of these proteins, 370 were detected in at least two samples of healthy leaves. A total of 313 proteins were common in at least two samples of healthy and PVA-infected leaves; of these proteins, 8 showed differential accumulation. Sixteen proteins were detected exclusively in the samples from PVA-infected leaves, whereas other 16 proteins were unique to healthy leaves. The protein Dnajc14 was only detected in healthy leaves, whereas different ribosomal proteins, ribosome-biogenesis proteins, and RNA splicing–related proteins were over-represented in the nuclei of PVA-infected leaves. Two virus-encoded proteins were identified in the samples of PVA-infected leaves.

**Conclusions:**

Our results show that PVA infection alters especially ribosomes and splicing-related proteins in the nucleus of potato leaves. The data increase our understanding of potyvirus infection and the role of nucleus in infection. To our knowledge, this is the first study of the nuclear proteome of potato leaves and one of the few studies of changes occurring in nuclear proteomes in response to plant virus infection.

## Background

Virus infections alter the homeostasis of plants, including that of gene expression, and suppress plant’s antiviral defenses, which enhance viral multiplication and spread throughout plant tissues. In this regard, analysis of plant-cell proteomes can shed light on the host pathways involved in the response/defense against virus attack, as well as pathways utilized by the virus to circumvent host defenses. Indeed, improvements in analytical methods have allowed the monitoring of changes in plant proteomes following virus infection [1–7].

Potyviruses (genus *Potyvirus*; Potyviridae) represent the largest and most economically damaging group of plant-infecting RNA viruses [8]. For example, potato virus A (PVA) and potato virus Y (PVY) infect potato (*Solanum tuberosum* L., family Solanaceae), which is the third most important food crop in the world, after rice and wheat. Yield losses up to 40% have been reported caused by PVA [9]. Potyviruses cause changes in the proteomes of entire cells as well as organelles, e.g. chloroplasts [10]. Dynamic changes in the transcriptome and proteome of potato leaves in response to infection with PVY strain NTN (PVY-NTN) have been compared between the potato cultivar Desiree and a transgenic line of this cultivar expressing salicylate hydroxylase, which catalyzes the NADH-dependent conversion of salicylate to catechol [4, 11]. The transcriptome analysis by Stare et al. [11] highlighted the dynamics of virus-induced changes, especially with respect to the regulation of light reactions– and sugar metabolism–related genes. Their analysis of potato leaf proteome revealed a total of 339 proteins that were mainly involved in photosynthesis, glycolysis, regulation of redox potential, post-translational modifications, RNA regulation and DNA synthesis [4]. Among those proteins, the cellular levels of 21 were altered in response to PVY infection. The differential proteins were found to be mainly involved in primary photosynthesis, but also in nitrogen metabolism, DNA synthesis, cofactor and vitamin metabolism, as well as protein synthesis, degradation and transport. Results of proteome and transcriptome analyses revealed no clear correlations [4].

Virus infection may affect subcellular localization of plant proteins and induce morphological changes in cell membranes [12, 13]. For example, several plant proteins, including translation eukaryotic initiation factor 4E (eIF4E), poly(A)-binding protein, heat-shock protein 70, and translation elongation factor 1A, are redistributed to potyviral 6 K2–induced membranous replication vesicles [14–17]. Similarly, the movement of potyviruses between host cells involves specific targeting of proteins to plasmodesmata at the plant cell wall, including virus-encoded cylindrical inclusion protein and P3N-PIPO protein [18].

RNA viruses that infect plants replicate in membranous structures in the cytoplasm. However, some of their proteins localize to the nucleus in virus-infected cells for unknown reasons [19]. For example, the RNA-dependent RNA polymerase (replicase) of potyviruses (also known as nuclear inclusion protein b, NIb) and nuclear inclusion protein a (NIa, the viral proteinase responsible for processing most of the proteolytic sites in the large potyviral polyprotein) are found in the plant-cell nucleus. Nuclear localization of NIa is controlled by the N-proximal part of the protein that contains a bipartite nuclear localization signal [20, 21]. The N-proximal portion of NIa encodes also the viral genome-linked protein (VPg) that is separated from NIa by a suboptimal cleavage site [20]. VPg interacts with fibrillarin in the nucleolus and Cajal bodies [21] and with ribosomal protein S6 kinase in the nucleus and nucleolus [22]. In addition, VPg and/or NIa recruits the plant poly(A) binding protein, DEAD-box RNA helicase–like protein, decapping protein 2 (DCP2), eIF4E and eIF(iso)4E to the nucleus [14, 15, 23–25]. Targeting of DCP2 to the nucleus inhibits formation of cytoplasmic DCP1/DCP2 granules, which may disrupt RNA decay –mediated degradation of turnip mosaic virus RNA [24].

An improved knowledge of the changes occurring in the plant-cell nuclear proteome during virus infection can be useful for understanding the role of the nucleus during the infection of RNA viruses. To our knowledge, however, only a single study on this topic has been published, reporting the nuclear proteome of hot pepper plants (*Capsicum annuum* L.) challenged with tobacco mosaic virus (TMV, genus *Tobamovirus*), which triggers a hypersensitive resistance response in pepper plants [26]. The nucleus of TMV-inoculated leaves was found to contain six proteins that were not found in the nucleus of mock-inoculated leaves of control plants [26].

The aim of this study was to carry out a comparative analysis of the nuclear proteomes between healthy and potyvirus-infected leaves of potato plants. Cultivated potato is a heterozygous autotetraploid having 48 chromosomes. To simplify our analysis, we used a diploid (2n = 2x = 24) interspecific potato line that is susceptible to PVA. Systemically infected leaves were harvested to obtain leaf samples with the highest proportion of PVA-infected cells. Results are expected to advance our knowledge about the role of the nucleus in virus infection, which in turn may inform new strategies to control virus infections in crops.

## Results

### Nuclear proteome of *S. tuberosum*

The pedigree of the diploid potato line v2–108 used in this study contained the *Solanum* species *phureja, tuberosum, chacoense*, *sparsipilum* and *stenotomum* [27, 28]. Three experiments were carried out with plants that had been systemically infected with PVA, and nuclear proteins were isolated from leaf cells. Nuclear proteins isolated from healthy potato leaves were included as controls. A total of 807 differential proteins were identified among the nuclear-protein samples of healthy and PVA-infected potato leaves using the reference genome sequence of *S. tuberosum* Group Phureja, clone DM1–3 (annotation v3.4) [29]. A protein was considered identified with false discovery rate (FDR) < 0.05 when two or more peptides, including at least one unique peptide, matched a known protein sequence.

**Fig. 1:**
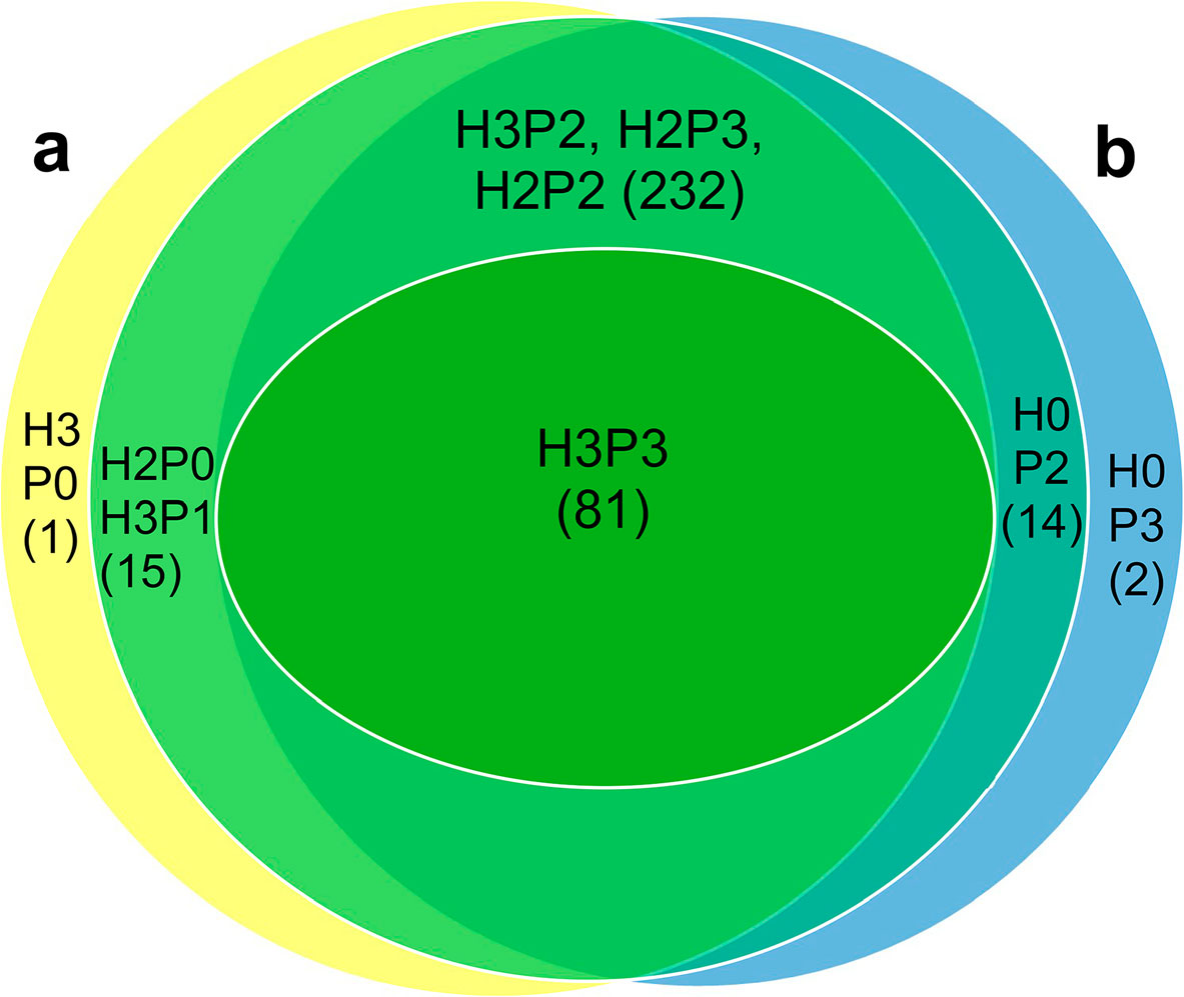
Grouping of nuclear proteins detected in PVA-infected and healthy potato leaves. The number of proteins identified in the nucleus of **a**, healthy control leaves and **b**, PVA-infected leaves is indicated in parenthesis. Abbreviations indicate: H, healthy; P, PVA-infected; 3, 2, 1 or 0, proteins detected, respectively, in three, two, one samples or not detected

The samples of nuclear proteins from healthy leaves resulted in the identification of 370 proteins that were in common among at least two out of the three experiments (Fig. 1a; Table S1), whereas 104 were detected in all three experiments. A higher number of proteins was identified in the experiments H2 and H3 as compared to the experiment H1. Among the proteins identified to be in common in two out of the three experiments, 93% were in common in the experiments H2 and H3 but missing from H1 (Table S1). The most abundant nuclear proteins in healthy potato leaves were various histones, such as H4, H2A and H2B, which represented 47% of the total spectral counts (Table S1). Additionally, several proteins of chloroplasts or the photosystem were detected, but these were likely contaminants acquired during the preparation of nuclear extract and so were not considered in the analysis. Different histone H1 and H3 proteins and nucleolin were common (Table S1), as expected. Histones H2A, H2B, H3 and H4 form the core of nucleosome, whereas histone H1 occupies the linker region connecting different nucleosomes. Other commonly detected proteins included ribosomal proteins and ribonucleoproteins, splicing-related proteins, stem 28-kDa glycoprotein (acid phosphatase 1-like), elicitor-inducible protein EIG-J7, phosphoglycerate kinase, subunits of glyceraldehyde-3-phosphate dehydrogenase, a matrix attachment region (MAR)-binding protein, an endoplasmin homolog, a homolog of fibrillarin, heat shock chaperones, eight isoforms of the 14–3-3 protein, and three ALY protein isoforms (Table S1).

**Fig. 2:**
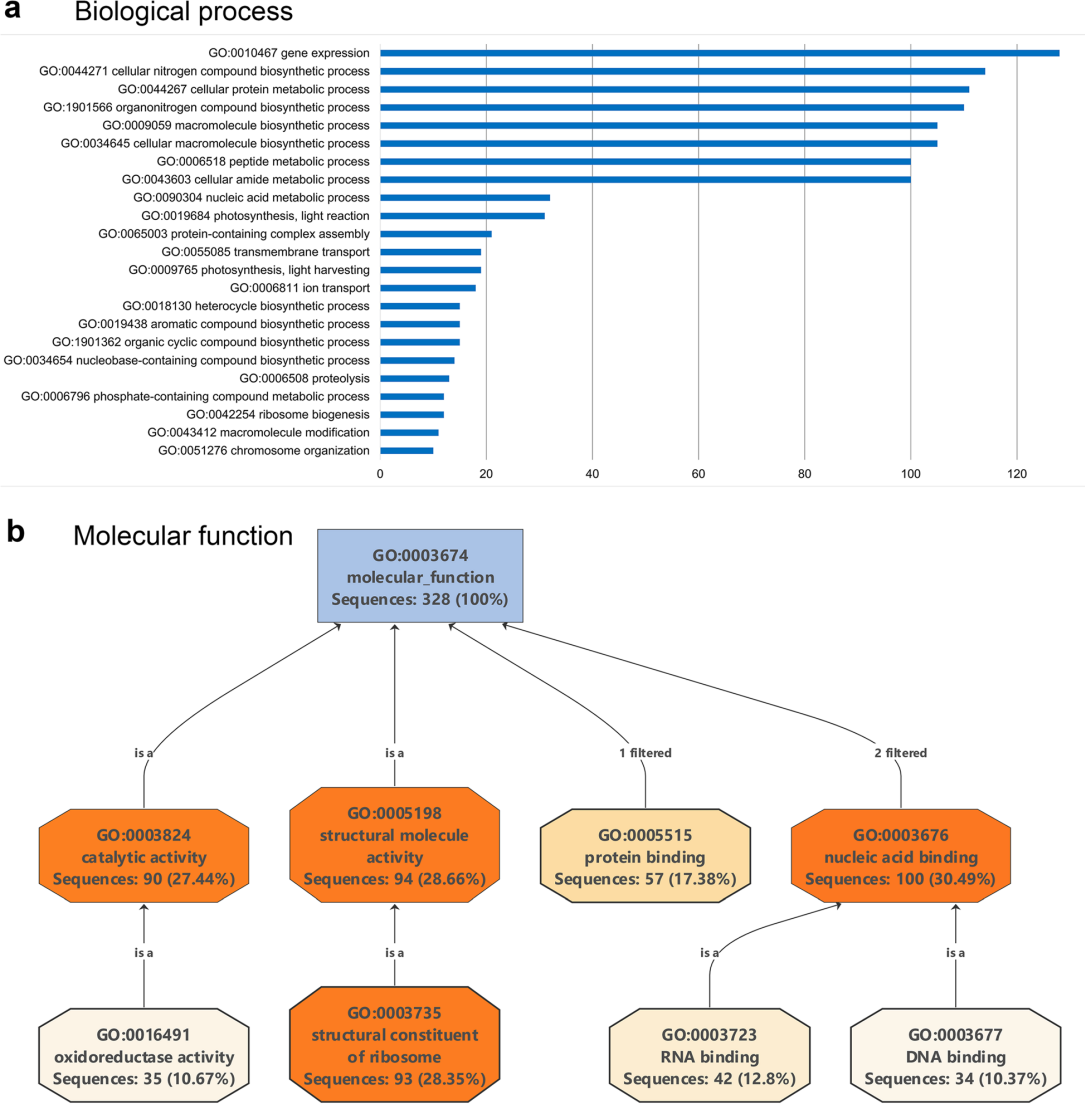
Functional annotation of the 451 proteins of the *S. tuberosum* nuclear proteome with Blast2GO analysis. **a**, Distribution of the detected proteins with respect to biological processes. Functional annotation at GO level 5 is shown. Note that the same protein may belong to different GO groups. Only processes that had a minimum of 10 proteins are shown. **b**, A combined GO annotation graph of molecular functions. Only those functions with 30 or more sequences are shown. Intermediate functions were excluded

Using Blast2GO software, the proteins were categorized according to their biological process, molecular function, and cellular localization. The nuclear proteome of potato leaves included proteins associated with, e.g., biological processes (GO level 2) such as organization of cellular components, biogenesis, response to various stimuli, and biological regulation and localization. Many of the identified proteins are involved in gene expression, translation, metabolic processes of peptides, and ribosome biogenesis (Fig. 2a). The proteins having a molecular function could be classified mainly as nucleic acid–binding or protein-binding proteins, structural constituents of ribosomes, or catalytic activities (Fig. 2b).

### Plant proteins in nuclei of PVA-infected cells

A total of 313 proteins were common in PVA-infected and healthy samples (groups H3P3, H3P2, H2P3, H2P2 in Fig. 1) and 81 were common in all nuclear protein samples (group H3P3 – three samples of PVA-infected leaves and three samples of healthy leaves; Fig. 1). Comparative proteomic analysis was carried out for the proteins that were detected common in healthy and PVA-infected samples (groups H3P3, H3P2, H2P3, H2P2 in Fig. 1) using the normalized spectral abundance factor - power law global error model (NSAF-PLGEM) [30]. Analysis indicated that eight proteins were differentially accumulated (Table 1). Six proteins (Kunitz trypsin inhibitor, ribosomal proteins L13, L18 and L27a, histone deacetylase complex subunit sap18-like protein and a spliceosomal protein) were induced in the presence of PVA, whereas levels of two proteins (Histone H2B and ribosomal protein L23) were decreased.

**Table 1:** Differences in the abundance of proteins that were detected common in PVA-infected and healthy samples (groups H3P3, H3P2, H2P3, H2P2, Fig. 1) as analysed using the normalised spectral abundance factor - power law global error model (NSAF-PLGEM)

Accession	Description (*Solanum tuberosum* Group Phureja DM1–3)^a^	Description (Blast2Go search)^b^	NSAF value^c^ (Healthy)			NSAF value^c^ (PVA-infected)			FDR^d^
			Score_H1	Score_H2	Score_H3	Score_P1	Score_P2	Score_P3	
PGSC0003DMP400024227	Kunitz trypsin inhibitor	miraculin-like	NA	0.0004	0.0008	0.0066	0.0038	0.0015	0.01
PGSC0003DMP400053772	60S ribosomal protein L13	60s ribosomal protein l13–1-like	NA	0.0020	0.0031	0.0214	0.0030	0.0029	0.02
PGSC0003DMP400052757	Histone H2B	probable histone H2B.3	0.1485	0.0481	0.0419	NA	0.0473	0.0380	0.03
PGSC0003DMP400062436	Ribosomal protein L23	60s ribosomal protein l23a-like	0.0093	NA	0.0012	NA	0.0008	0.0011	0.03
PGSC0003DMP400031685	Ribosomal protein L27a	60s ribosomal protein l27a-3-like	NA	0.0008	0.0008	0.0049	0.0015	0.0011	0.05
PGSC0003DMP400024215	P18	histone deacetylase complex subunit sap18-like	NA	0.0016	0.0008	0.0082	0.0008	0.0011	0.05
PGSC0003DMP400042811	60S ribosomal protein L18	60S ribosomal protein L18–2-like	NA	0.0020	0.0027	0.0082	0.0057	0.0033	0.05
PGSC0003DMP400052999	Spliceosomal protein	U2 small nuclear ribonucleoprotein B″	0	0.0016	0.0012	0.0082	0.0019	0.0015	0.05

^a^Description (*Solanum tuberosum* Group Phureja DM1–3 Genome Annotation v3.4)

^b^Description (Blast2GO search against protein sequences)

^c^**N**ormalized **S**pectral **A**bundance **F**actor for the protein. Score_H1-H3 and Score_P1-P3 indicate values for each three experiment. NA, not applicable

^d^False discovery rate value

Sixteen proteins were unique to PVA-infected leaves (Table 2). Among these proteins, two were detected in all three PVA samples (group H0P3; Fig. 1), and 14 proteins were detected in two of the three PVA samples (group H0P2; Fig. 1). However, all these 16 proteins were absent from all three healthy samples.

**Table 2:** Proteins unique for the nuclear samples of PVA-infected potato leaves

Accession	Description (*Solanum tuberosum* Group Phureja DM1–3)^a^	Description (Blast2GO search)^b^	PSM value^c^ (PVA-infected)			PSM value^c^ (healthy)	Group^d^
			Score_P1	Score_P2	Score_P3		
PGSC0003DMP400020209	Pre-mRNA-splicing factor SF2	serine arginine-rich-splicing factor sr34-like isoform ×1	3	5	1	0	H0P3
PGSC0003DMP400037788	60S ribosomal protein L8	60S ribosomal protein L8	1	2	2	0	H0P3
PGSC0003DMP400014216	60S ribosomal protein L12	60S ribosomal protein L12	0	8	8	0	H0P2
PGSC0003DMP400046977	Small nuclear ribonucleoprotein E	small nuclear ribonucleoprotein E-like	7	6	0	0	H0P2
PGSC0003DMP400025902	Glycoprotein	60S ribosomal protein L14–1	3	8	0	0	H0P2
PGSC0003DMP400012614	60s acidic ribosomal protein	60S acidic ribosomal protein P2-like	2	3	0	0	H0P2
PGSC0003DMP400043902	Ribosomal protein S26	40S ribosomal protein S26–3-like	0	3	2	0	H0P2
PGSC0003DMP400047103	Ferritin	ferritin-2, chloroplastic	0	3	2	0	H0P2
PGSC0003DMP400054155	Glutathione S-transferase	probable glutathione S-transferase	0	3	2	0	H0P2
PGSC0003DMP400010248	RRNA-processing protein UTP23	rRNA-processing protein UTP23 homolog isoform X1	2	2	0	0	H0P2
PGSC0003DMP400043895	Splicing factor 3B subunit	Splicing factor 3B subunit 5/RDS3 complex subunit 10	0	2	1	0	H0P2
PGSC0003DMP400007597	Serine/arginine-rich protein	serine/arginine-rich SC35-like splicing factor SCL33	1	2	0	0	H0P2
PGSC0003DMP400017980	HCF106	sec-independent protein translocase protein TATB, chloroplastic	0	1	2	0	H0P2
PGSC0003DMP400049837	Microsomal signal peptidase 23 kDa subunit	signal peptidase complex subunit 3B	0	1	2	0	H0P2
PGSC0003DMP400015542	Small GTP-binding protein	ras-related protein RABE1a-like	0	1	2	0	H0P2
PGSC0003DMP400027641	60S ribosomal protein L13a	60S ribosomal protein L13a-4	1	0	2	0	H0P2

^a^Description (*Solanum tuberosum* Group Phureja DM1–3 Genome Annotation v3.4)

^b^Description (Blast2GO search against protein sequences)

^c^The number of **P**eptide **S**pectrum **M**atches (the total number of identified peptides matched for the protein). Score_P1-P3 indicate values for each three experiment

^d^H0P3, proteins detected in all three PVA samples but no healthy sample; H0P2, proteins detected in two of three PVA samples but no healthy sample

The proteins that were unique to three PVA samples (and absent from all healthy leaves) were pre-mRNA-splicing factor SF2 and 60S ribosomal protein L8 (Table 2, group H0P3). Proteins that were unique to two PVA samples (and absent from all healthy samples) included five ribosomal proteins (RPL12, RPL13a, RPL14, RPS26, P2-like), two pre-mRNA splicing–related proteins (splicing factor 3B subunit and a plant-specific serine/arginine-rich protein), small ribonuclear protein E, glutathione-S-transferase, rRNA processing protein UTP23, a ras-related small GTPase protein, ferritin, sec-independent protein translocase HCF106, and a microsomal signal peptidase subunit (Table 2; group H0P2). Besides the ribosomal and splicing-related proteins mentioned above, small ribonuclear protein E is also involved in pre-mRNA splicing. The rRNA processing protein UTP23 is involved in rRNA processing and ribosome biogenesis. These results demonstrated that proteins associated with ribosomes and pre-mRNA splicing were abundant among the proteins exclusively found in PVA samples.

In addition to the genome sequence of *S. tuberosum* Group Phureja (clone DM1–3) [29], the MS/MS peptide data were subjected to a search of the NCBI genome annotation of *S. tuberosum* (release 100). The database search identified 17 proteins found exclusively in the PVA samples, one of which was splicing factor 3B–related protein that was found in all three PVA samples. The other 16 proteins were detected in two out of three PVA-infected samples but were missing in all healthy samples (Table S2). Eleven proteins matched or were homologs of proteins that were unique in PVA-infected samples (*S. tuberosum* Group Phureja DM1–3). Three proteins were previously unknown: a RPS27–2-like protein, lysine-specific demethylase/transcription factor, and the sec-independent protein translocase protein TATB (Table S2).

### Plant proteins that were absent from the nucleus of PVA-infected cells

Eleven proteins were found exclusively in the healthy samples (Table 3). One protein (Dnajc14 protein) was present in all three healthy samples but was missing from all PVA-infected samples (Table 3, group H3P0). Ten proteins, including two chloroplast proteins, were found in two healthy samples (group H2P0 in Fig. 2; Table 3) but were absent from all PVA-infected samples. As viruses are unevenly distributed in leaves, the group H3P1 (Fig. 1; Table 3) including five proteins found in common to all three healthy samples, but present only in one PVA-infected sample, may be also noteworthy. The proteins missing in all or most of the PVA-infected samples included a variant of histone H2B, calreticulin, pinin/SDK/memA protein, knotted-like homeobox protein, splicing factor 3b subunit, four small nuclear ribonucleoproteins, elongation factor P, two 60S ribosomal proteins (RPL12 and RPL14) and snakin-2 (Table 3).

**Table 3:** Proteins unique for the nuclear samples of healthy potato leaves

Accession	Description (*Solanum tuberosum* Group Phureja DM1–3)^a^	Description (Blast2Go search)^b^	PSM value^c^ (healthy)			PSM value^c^ (PVA-infected)	Group^d^
			Score_H1	Score_H2	Score_H3		
PGSC0003DMP400016511	Dnajc14 protein	homeobox protein Hox-A10	1	2	1	0	H3P0
PGSC0003DMP400012698	Histone H2B	histone H2B	48	102	0	0	H2P0
PGSC0003DMP400040333	Calreticulin	calreticulin-3-like isoform X1	0	8	6	0	H2P0
PGSC0003DMP400051884	Pinin/SDK/memA protein	pinin	0	4	4	0	H2P0
PGSC0003DMP400027372	Class II knotted-like homeobox protein	homeobox protein knotted-1-like 3 isoform X2	0	4	3	0	H2P0
PGSC0003DMP400048828	Splicing factor	splicing factor 3B subunit 1	0	4	2	0	H2P0
PGSC0003DMP400022318	RNA binding protein	heterogeneous nuclear ribonucleoprotein 1	1	0	5	0	H2P0
PGSC0003DMP400026205	U1 small nuclear ribonucleoprotein 70 kDa	U1 small nuclear ribonucleoprotein 70 kDa	2	0	1	0	H2P0
PGSC0003DMP400054431	Elongation factor P (EF-P)	elongation factor P	0	1	2	0	H2P0
PGSC0003DMP400031700	Elongation factor TuA, chloroplastic	elongation factor TuA, chloroplastic	0	6	7	0	H2P0
PGSC0003DMP400022105	PGR5 1A, chloroplastic	PGR5-like protein 1B, chloroplastic	0	2	2	0	H2P0
PGSC0003DMP400006029	60S ribosomal protein L12	60S ribosomal protein L12	2	5	8	4	H3P1
PGSC0003DMP400034967	Glycoprotein	60S ribosomal protein L14–1	2	3	7	6	H3P1
PGSC0003DMP400044310	Small nuclear ribonucleoprotein polypeptide	small nuclear ribonucleoprotein E-like	1	7	2	2	H3P1
PGSC0003DMP400002893	Snakin-2	snakin-2	1	1	6	8	H3P1
PGSC0003DMP400034062	Nucleic acid binding protein	RNA-binding protein 2-like isoform X1	2	1	1	5	H3P1

^a^Description (*Solanum tuberosum* Group Phureja DM1–3 Genome Annotation v3.4)

^b^Description (Blast2GO search against protein sequences)

^c^The number of **P**eptide **S**pectrum **M**atches (the total number of identified peptides matched for the protein). Score_H1-H3 indicate values for each three experiment. In PVA-infected samples, the positive value is for Score_P1 or Score_P3. The other scores were zero

^d^H3P0, proteins detected in all three healthy samples but no PVA sample; H2P0, proteins detected in two of three healthy samples but no PVA sample; H3P1, proteins detected in all three healthy samples and in one PVA sample

Nuclear proteins were also identified by comparison with the peptides and proteins in the NCBI genome annotation of *S. tuberosum* release 100. The search identified 14 proteins that were exclusively found in healthy potato leaves: Dnajc14 was present in all three samples (the same finding was made in the peptide search of *S. tuberosum* Group Phureja), and seven proteins were found in two samples. Six proteins were found in all three healthy samples and in one PVA-infected sample (Table S3). Half of all proteins were homologs of proteins expressed in *S. tuberosum* Group Phureja, but there were also newly identified proteins including brefeldin A, a resistance-like protein, and a predicted calmodulin binding protein and a bicaudal C homolog 1–like protein (Table S3).

**Fig. 3:**
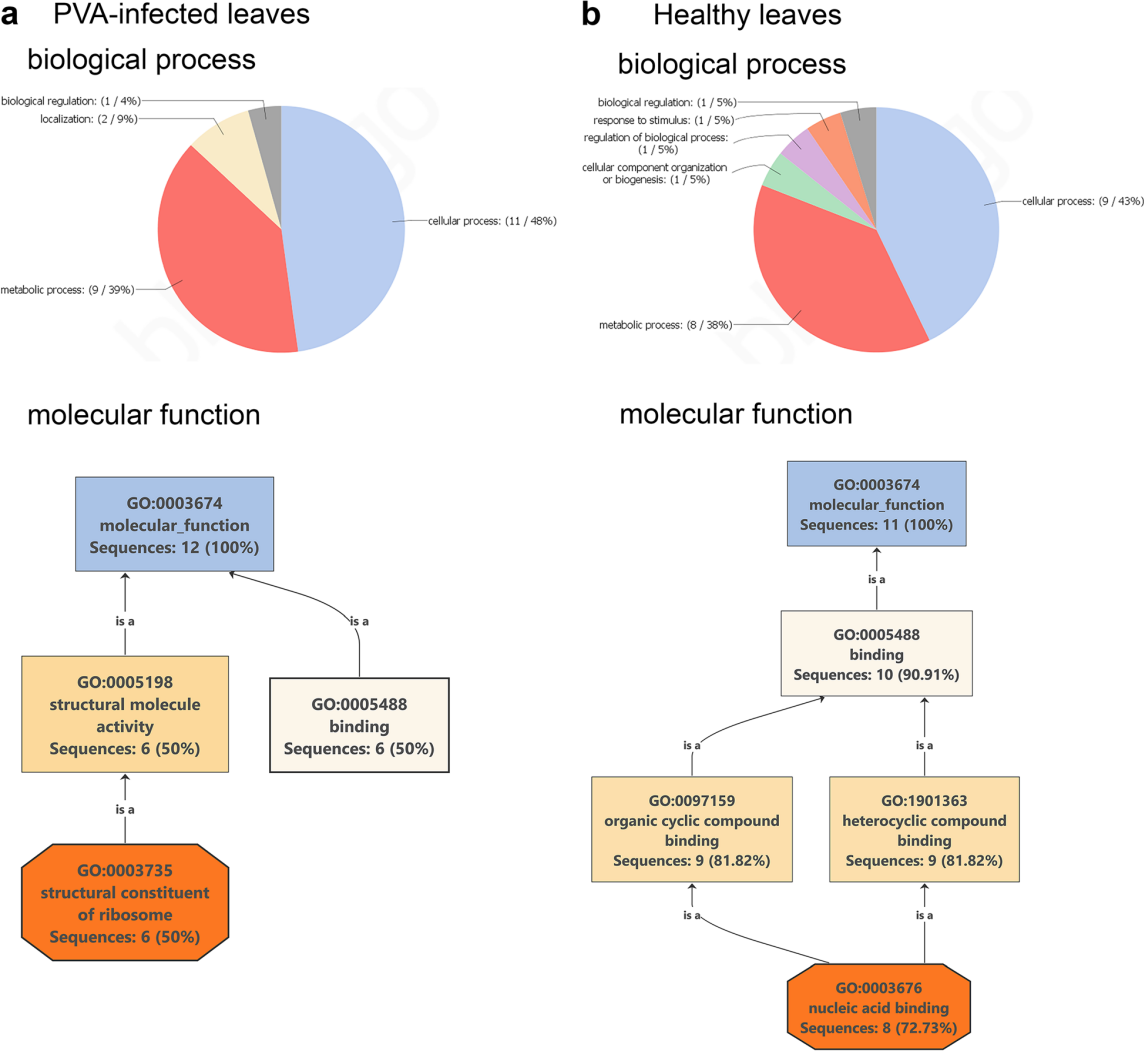
Distribution of nuclear proteins with respect to the GO categories “biological processes and molecular functions”. Included were nuclear proteins that appeared or disappeared in response to PVA infection. **a**, Proteins newly appearing in response to PVA infection. Biological processes are presented at GO level 2 in the pie chart. Only molecular functions with five or more sequences are shown. **b**, Proteins that were absent after PVA infection. Biological processes are presented at GO level 2 in the pie chart. Only molecular functions with five or more sequences are shown. Note that the same protein may be categorized in several GO classes

To predict the biological processes and molecular functions controlled by the nuclear proteins, we carried out a Blast2GO analysis of the proteins that were present or absent following PVA infection and were identified in the search of *S. tuberosum* Group Phureja. Proteins appearing in response to PVA infection belonged to biological processes (GO level 2), such as cellular or metabolic processes, localization, and biological regulation. More specifically, the detected proteins were found to be involved in especially gene expression, metabolism, and biosynthesis of macromolecules and peptides, and translation. The main molecular functions were associated with binding or structural constituents of ribosomes (Fig. 3a). On the other hand, proteins that disappeared in response to PVA infection represented cellular or metabolic processes (especially gene expression), organization of cellular components, biogenesis, regulation of biological processes, and responses to various stimuli. The main molecular functions were dominated by proteins involved in the binding of nucleic acids (Fig. 3b).

### Viral proteins detected in the nuclear proteome of virus-infected leaves

**Fig. 4:**
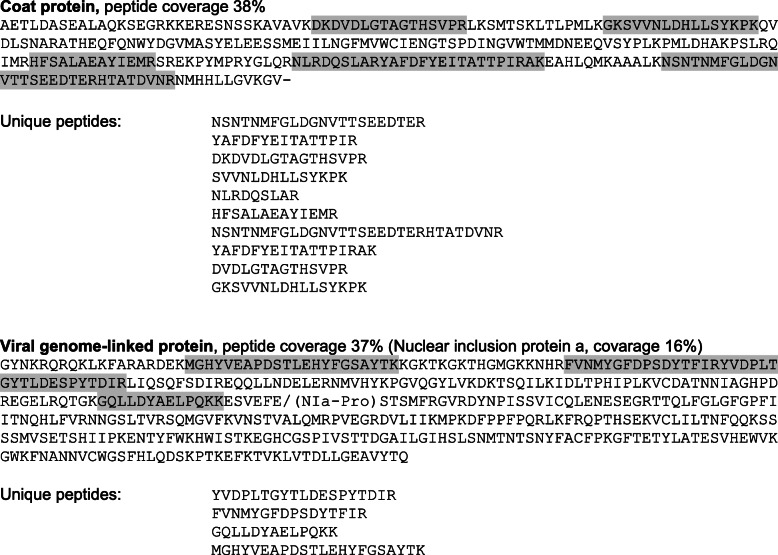
Amino acid sequences of PVA coat protein (CP) and nuclear inclusion protein (NIa) showing the peptide coverage determined by the nuclear proteome. The identified peptides are shown in bold and are underlined. The backslash within the NIa sequence indicates the suboptimal proteolytic cleavage site, which separates the N-terminal viral genome-linked protein (VPg) and the C-terminal protease domains of NIa. All identified peptides of NIa (coverage 16%) are located in the VPg domain (coverage 37%). Unique peptides are listed separately for each of the two proteins

Besides plant proteins, peptides specific to two virus-encoded proteins were detected in PVA-infected samples but were absent from healthy samples. The identified proteins were CP, NIa, and VPg of PVA. Based on spectral counting, CP was the most abundant viral protein, followed by VPg. CP was identified based on 10 unique peptides with peptide coverage of 38% (Fig. 4). NIa consists of two domains: the N-proximal VPg that contains a strong bipartite nuclear localization signal [21], and a C-proximal proteinase. Four unique peptides were associated with NIa. All peptides were derived from the VPg region of the PVA polyprotein (Fig. 4).

### Localization of a ras-related small GTPase protein in cells of healthy and PVA-infected leaves

**Fig. 5:**
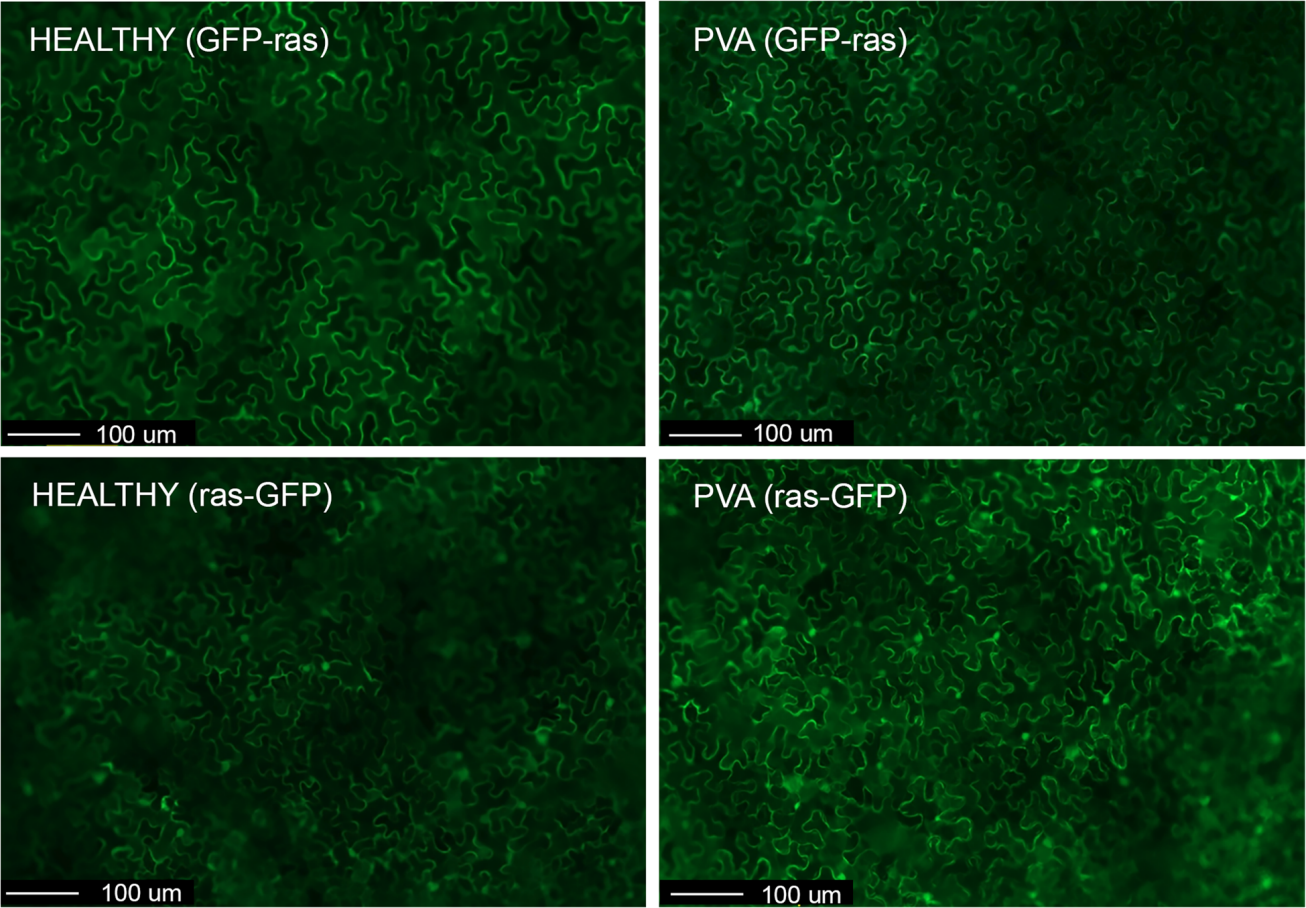
Subcellular localization of the GFP-rabE1a and rabE1a-GFP fusion proteins in healthy and PVA-infected leaves of *N. benthamiana* at 3 days after agroinfiltration. GFP-ras and ras-GFP are shown for comparison. Scale bars = 100 μm

One of the proteins found uniquely in the nucleus of PVA-infected cells was Rab-like GTPase (Table 2). Rab GTPases play central roles in endomembrane trafficking [31]. Viruses exploit host membrane trafficking pathways and previous studies implicate a role for Rab GTPases in virus infection [32–35]. A Rab-like GTPase has been also found induced in the nuclei of hot pepper plants in response to TMV infection [26]. Therefore, the ras-related small GTPase protein rabE1a was cloned as a fusion with GFP to verify its localization in healthy and PVA-infected cells. In order to identify the cells infected with PVA, we used PVA tagged with RFP (PVA-RFP). Leaves of healthy *Nicotiana benthamiana* plants and leaves systemically infected with PVA-RFP were subjected to agroinfiltration with the GFP fusion proteins, and the localization of the fusions was assessed. In healthy leaves, GFP-rabE1a was mainly observed in the cytoplasm (98.8% of analyzed cells), whereas in the PVA-infected cells GFP-rabE1a was detected in both the cytoplasm and nucleus (36.7%) or cytoplasm only (63.3%) (Table 4, Fig. 5). In the case of rabE1a-GFP (i.e., C-terminal tag), fluorescence was detected in the cytoplasm and nucleus of many cells and no differences were observed between healthy and PVA-infected cells (Table 4).

**Table 4:** Distribution of fluorescence from GFP-rabE1a/rabE1a-GFP in the cytoplasm and nucleus of healthy and PVA-infected cells

Construct	Number of analyzed cells^a^	Fluorescence in both cytoplasm and nucleus	Percentage (%) of cells with nuclear fluorescence^b^
GFP-rabE1a (healthy)	257	3	1.2
GFP-rabE1a (PVA-infected)	283	104	36.7
rabE1a-GFP (healthy)	236	120	50.8
rabE1a-GFP (PVA-infected)	297	108	36.4

^a^Total number of counted cells that showed fluorescence

^b^Percentage of cells that showed fluorescence both in the cytoplasm and nucleus. In all other cells, fluorescence was detected only in the cytoplasm

## Discussion

Several proteomics studies have been carried out among members of the family Solanaceae but only few have characterized nuclear proteome [36]. Characterization of tomato (*Solanum lycopersicum* cultivar Ailsa Craig) nuclear proteome during fruit ripening process showed prevalence of proteins involved in gene regulation, translation, proteolysis, structure, and detoxification [37]. Furthermore, analysis of the nuclear proteome of tomato after infection with the oomycete *Phytophthora capsici* revealed dynamic changes in nuclear protein composition, including proteins involved in immunity [38]. In this study, the nuclear proteome of potato was analyzed for the first time. We identified 370 nuclear proteins in healthy leaves of the diploid potato line v2–108 and 40 proteins accumulated differently in response to PVA infection. The most abundant proteins identified in both healthy and PVA-infected nuclear proteomes of potato leaves included proteins involved in chromatin assembly and remodeling (variants of histones H2A, H2B, H3, H4 and H1) and histone deacetylases. In addition, we identified a large proportion of translation-related proteins, including various ribosomal proteins, in both healthy and PVA-infected samples. These results resembled those of the nuclear proteome of rice, which contains a large proportion of histone proteins [39, 40]. However, our results on the nuclear proteome of potato differ from those observed in *Arabidopsis thaliana* (L.) Heynh. and chickpea (*Cicer arietinum* L.), where relative predominance of proteins related to signaling and gene regulation was observed [41, 42]. We found also in the nuclear proteome of potato several proteins homologous to the nucleolar proteome of *A. thaliana* [43].

In the nuclear proteome of PVA-infected potato leaves we identified several ribosomal proteins and proteins involved in ribosome biogenesis that were absent or less accumulated in the nuclear proteome of healthy potato leaves. Previous studies showed that potyvirus infections increase the expression of a set of protein synthesis–related genes, particularly the genes encoding ribosomal proteins at the transcriptional level [44–47]. Thus, a large number of ribosomal proteins identified in the nuclei of PVA-infected leaves is consistent with the transcriptome data.

In plants, ribosomal proteins are encoded by small multigene families, and functional specialization of family members has been related to multiple copies of individual ribosomal proteins [48, 49]. Several ribosomal protein mutants have specific phenotypes, which may suggest that the composition of ribosomes plays an important role in plant development [48]. Differential induction of ribosomal protein family members (e.g., RPL13) during infection of *A. thaliana* with turnip mosaic virus (genus *Potyvirus*) has also been documented [45]. Some minor changes in ribosome populations have been detected when the riboproteomes of healthy, *Agrobacterium tumefaciens*–infected and PVA-infected leaves of *N. benthamiana* were compared [50]. Ribosomal proteins mainly reside on the surface of ribosomes, whereas rRNA molecules form the core of the complex and may directly interact with the mRNA [48]. Thus, ribosome heterogeneity has been speculated to reflect functional diversity [49]. In support of this, ribosomes lacking certain ribosomal proteins preferentially translate leaderless mRNAs in *Escherichia coli* [51], whereas ribosomes lacking ribosomal protein S25 are defective in translation of certain viral mRNAs [49, 52]. In our study, we found alterations in the cellular abundance of translation-related proteins, which suggests that PVA induces a general reprogramming of plant-cell metabolism and also proposes that potyviruses may modify the composition of ribosomes to enhance the translation of virus-encoded RNAs.

Certain ribosomal proteins also have functions outside ribosomes. They may regulate host gene transcription, modulate the activities of transcriptional regulators, or control mRNA translation external to ribosomes [53]. For example, RPL22 of *Drosophila melanogaster* associates with linker (histone H1) and co-localizes with condensed chromatin, and its overexpression suppresses transcription [54]. In mammals, RPL13 controls translation of mRNAs having a specific structure in the 3′ non-translated region [55]. Phosphorylated RPL13a is released from the ribosome and binds to eIF4G, which prevents the entry of the 43S ribosomal complex [55]. In plants, silencing of RPL13 reduces the efficiency of potyvirus infection [56]. In our study, a variant of RPL13 was identified in the nuclear proteome of PVA-infected leaves but not of healthy leaves. In addition, another variant of RPL13 was induced in the presence of PVA, which also suggests a role for RPL13 in virus infection.

The second class of proteins that were found to accumulate differently in the nuclei of PVA-infected leaves versus healthy leaves comprised proteins related to pre-mRNA splicing. Three splicing factors and one splicing-related protein were unique in PVA samples and were not found in healthy samples. Two splicing-related proteins were detected uniquely in healthy samples and one splicing-related protein showed differential accumulation. Various abiotic and biotic stresses may affect alternative splicing in plants [57, 58]. Alternative splicing is a major mechanism by which proteome diversity is enhanced [59]. Splicing factors guide the spliceosomal complex to splice sites of pre-mRNAs and determine which splice sites are selected under each condition. Many genes that encode proteins with regulatory functions, including splicing factors, and genes involved in the response of plants to stress are targeted for alternative splicing [58, 59] that may be induced or modulated by different stresses. Also, several plant resistance genes are regulated by alternative splicing, and the different protein forms are needed to establish full plant resistance [58]. Accumulation of different splicing-related genes in the nuclei of PVA-infected versus healthy leaf cells may suggest that potyvirus infection—or virus infection in general—affects alternative splicing in plants. In support of this, extensive changes in alternative splicing in plants of *Brachypodium distachyon* infected with panicum mosaic virus (genus *Panicovirus*) and its satellite virus have been documented as compared with healthy plants using high-throughput RNA sequencing [60].

One protein that was found uniquely in the nuclear proteome of PVA-infected leaves was a Rab-like GTPase protein (rabE1). This protein may have an important role in intracellular vesicle trafficking including exocytosis and endocytosis [31]. Accordingly, a Rab-like GTPase was found to be induced in nuclei of TMV-infected hot pepper as compared with control plants [26]. Plant viruses induce membrane remodeling, and membrane trafficking is essential for virus infection [12, 13]. Previous studies showed a role for different Rab GTPases in intracellular movement and replication of animal and plant viruses [32–35]. Differential subcellular localization of a Rab-like GTPase (rabE1a) found in our study was confirmed in PVA-infected and healthy leaves by analyzing the subcellular localization of a GFP-tagged fusion. The protein was detected both in the nucleus and cytoplasm of PVA-infected cells but essentially in the cytoplasm of healthy cells. Thus, the subcellular localization data are consistent with the results obtained with the nuclear proteomics. The difference in subcellular localization was obvious when GFP-rabE1a was used, whereas rabE1a-GFP was found in both the cytoplasm and nucleus in both healthy and PVA-infected cells. Rab-like GTPases typically localize to intracellular membranes [61] and a conserved, C-terminal prenylation motif mediates the targeting [62]. Thus, C-terminally tagged rabE1a was probably mislocalized, similarly to C-terminally tagged yeast ras2 protein, which is found in the nucleus and cytoplasm, whereas the N-terminally tagged version localizes into intracellular membranes [63].

The potyvirus-encoded proteins NIa and NIb accumulate mainly in the nuclei of virus-infected cells [20, 21, 64, 65]. Several unique peptides specific to the VPg domain of NIa were detected in the nuclear protein samples of PVA-infected leaves, but the peptides were absent from the samples of healthy leaves. These data indicate that VPg/NIa is present in the nuclear proteome of PVA-infected potato leaves, as expected. Surprisingly, no peptides specific to NIb were found, even if both NIa and NIb have been reported to accumulate in the nucleus in large amounts. They may also form nuclear inclusions [64, 66, 67]. It is possible that NIb was present in the nuclei of PVA-infected leaves at levels too low to be detected, e.g., because of rapid degradation or its association with aggregates of nuclear inclusions.

Several unique peptides specific to PVA CP were also detected. Localization of potyviral CP to the nucleus has not been reported, except in a study that found particles of PVY (genus *Potyvirus*) near the nuclear pore complex and inside the nucleus [68]. Hence, CP may reside in the nucleus under certain cellular conditions. However, it may represent also chloroplast contaminants of nuclear extracts.

## Conclusions

We have characterized, for the first time, the nuclear proteome of potato and analyzed changes that occur in this proteome during potyvirus infection. The data indicate that potyvirus infection particularly affects ribosomes and splicing-related proteins in the nucleus. These proteins are thus interesting targets for further studies and analysis to understand more specifically how virus infection alters translation and/or pre-mRNA splicing.

## Methods

### Plant material

PVA-susceptible potato line v2–108 was propagated as previously described [69]. The line is a diploid (2n = 2x = 24) F1 hybrid, and its pedigree includes *Solanum tuberosum subsp. andigena*, *S. tuberosum* subsp. *tuberosum*, *S. chacoense*, *S. phureja*, *S. sparsipilum* and *S. stenotomum* [27, 28]. Plants were multiplied by rooting stem cuttings for subsequent growth in a growth chamber [photoperiod 16 h, light intensity 110 μE m^− 2^ s^− 1^, temperature 22–24 °C (light), 18–20 °C (dark), relative humidity 40%]. Fertilizer (N:P:K = 16:9:22, Yara, Finland) was mixed in water (0.3 g/L) and applied at each watering.

### Virus inoculation and detection

Potato plants were inoculated mechanically with sap extracted from leaves of *Nicotiana benthamiana* infected with the infectious clone PVA-BUIII [70]. For detection of systemic infection, the leaflets in the upper non-inoculated leaves were tested 20 days post-inoculation by double-antibody sandwich enzyme-linked immunosorbent assay (ELISA) using monoclonal antibody 58/0 specific for PVA coat protein (CP; SASA, Edinburgh, UK) along with an alkaline phosphatase–conjugated monoclonal secondary antibody, as described [71]. Leaf tissue from one side of the mid-rib was excised from each leaflet with a scalpel, weighed, and ground in ELISA sample buffer at 1 g per 3 mL. Aliquots (100 μL) were transferred to a microtiter plate coated with anti-PVA-CP. The other half of the leaflet was saved for isolation of nuclei, provided that the leaflet was PVA-positive. Leaflets from mock-inoculated plants were used as negative controls for ELISA and for isolation of nuclei for comparison of the nuclear proteomes between PVA-infected and healthy leaves.

### Isolation of nuclei, and protein extraction

**Fig. 6:**
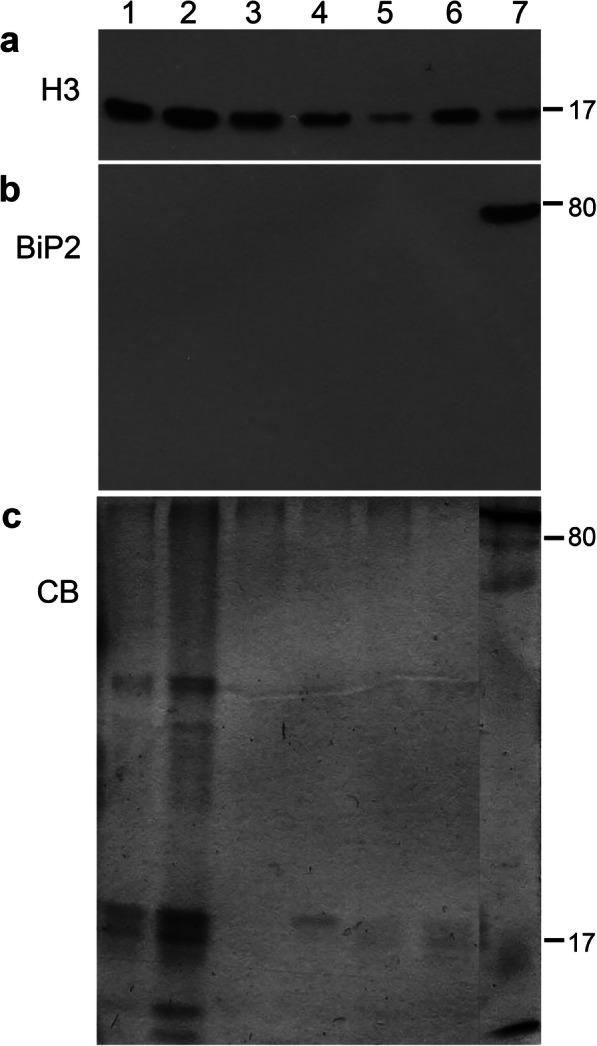
Analysis of nuclear proteins in healthy and PVA-infected potato leaves (line v2–108) used for shotgun proteomics. **a**, Histone H3 and **b**, luminal-binding protein 2 (BiP2) were detected using specific antibodies. **c**, Coomassie blue (CB) staining of the gel. Lanes: 1, healthy experiment-1; 2, PVA-infected experiment-2; 3, healthy experiment-3; 4, PVA-infected experiment-3; 5, healthy experiment-2; 6, PVA-infected experiment-1; 7, total protein fraction

Four PVA-positive potato leaflets (total weight 3–4 g), each from a different plant, were combined for the preparation of nuclear proteins. Leaflets taken from equivalent positions of mock-inoculated healthy potato plants served as controls. Nuclear extract was prepared using our protocol optimized for potato leaves [69]. Nuclear proteins were extracted using TRIzol reagent (Invitrogen, Carlsbad, CA). Proteins were precipitated with acetone, resuspended in 6 M urea, and analyzed by 12% SDS-PAGE followed by silver staining. Each nuclear-protein preparation was checked by western blotting for the presence of nuclear proteins (histone protein H3) and the absence of non-nuclear proteins (luminal-binding protein 2, known as BiP2) (Fig. 6, Fig. S1), as described [69].

### Mass spectrometry

The concentration of bulk nuclear proteins in samples was adjusted to be comparable based on A260 and A280 measurements. Cysteine bonds of proteins were reduced by treatment with 50 mM Tris (2-carboxyethyl) phosphine hydrochloride (Sigma-Aldrich, St. Louis, MO) at 37 °C for 20 min, and the reduced adducts were alkylated with 0.15 M iodoacetamide (Sigma-Aldrich) at room temperature. Samples were digested by the addition of 1 μg trypsin (Sequencing Grade Modified Trypsin, Promega). The resulting peptide fragments were purified with a C18 microspin column (Harvard Apparatus, Holliston, MA) and dissolved in 30 μL of buffer (0.1% trifluoroacetic acid and 1% acetonitrile in mass spectrometry–grade water). Liquid chromatography–coupled to tandem mass spectrometry (LC-MS/MS) of the peptide fragments was carried out with an EASY-nLCII system (Thermo Fisher Scientific, Wilmington, DE) connected to a Velos Pro-Orbitrap Elite ETD hybrid mass spectrometer (Thermo Fisher Scientific) with a nanoelectrospray ion source using the Xcalibur version 2.2 SP 1.48 (Thermo Fisher Scientific). The samples were separated using a two-column LC system consisting of a 2-cm C18-A1 trap column (Thermo Fisher Scientific) followed by a 10-cm C18-A2 analytical column (EASY-Column 10 cm × 75 μm, 3 μm, 120 Å; Thermo Fisher Scientific). The elution buffers were: buffer A, 0.1% formic acid, 0.01% trifluoroacetic acid and 1% acetonitrile in HPLC grade water; buffer B, 0.1% formic acid and 0.01% trifluoroacetic acid in 98% acetonitrile. The program for separation gradient consisted of 5% buffer B for 5 min, 35% buffer B for 60 min, 80% buffer B for 5 min, and 100% buffer B for 10 min. The flow rate was 0.3 μL/min. A single sample (4 μL) was injected per LC-MS/MS run. The analyses were performed in data-dependent acquisition mode using collision-induced dissociation. A full MS scan was acquired with a resolution of 60,000 at normal mass range in the Orbitrap mass spectrometer. The method was designed to fragment the 20 most intense precursor ions produced by collision-induced dissociation (energy 35 eV). Data were acquired using using the Xcalibur version 2.7.0 SP1 (Thermo Fisher Scientific).

### Protein data analysis

Data acquired from LC-MS/MS (i.e., peak files from the Orbitrap Elite) were used to identify the corresponding proteins using Proteome Discoverer software with SEQUEST search algorithm (version 1.4, Thermo Fisher Scientific). The search was done against the sequence of *S. tuberosum* Group Phureja DM1–3 genome (PGSC_DM_v3.4_pep_non-redundant, 52,925 proteins, http://solanaceae.plantbiology.msu.edu/pgsc_download.shtml) [29, 72]. In addition, the LC-MS/MS data were searched against the protein data of *S. tuberosum* at the National Center for Biotechnology Information (NCBI; *S. tuberosum* Annotation Release 100; ftp://ftp.ncbi.nlm.nih.gov/genomes/Solanum_tuberosum/protein/). A mass error of 15 ppm was allowed for the precursor ions and for the fragment in 0.8 Da. A static residue modification parameter was set for the carbamidomethyl + 57.021 Da (C) of cysteine residues. Methionine oxidation was set as a dynamic modification of + 15.995 Da (M). Only full-tryptic peptides were considered for scoring, and a maximum of one missed cleavage was allowed. The peptide false discovery rate (FDR) was set to < 0.05. In addition, only proteins with two or more peptide matches that included at least one unique peptide were considered as having been identified.

PVA-infection induced differences in the abundance of proteins that were detected common in PVA-infected and healthy samples (groups H3P3, H3P2, H2P3 and H2P2) were compared in R using the Bioconductor package PLGEM version 1.60.0. The normalized spectral abundance factor (NSAF) counts were calculated for each identified protein from raw spectral counts and they were used as input values for PLGEM. Differentially expressed proteins were identified via a permuted signal-to-noise test statistics [30]. Blast2GO version 5.2.5 (http://www.blast2go.org) was used to categorize the proteins detected by Gene Ontology (GO) annotation according to their biological process, molecular function, and cellular component (http://www.geneontology.org). Protein domains were detected using Blast2GO InterProScan tool and searched at Pfam website (http://pfam.sanger.ac.uk).

### Construction of plasmids encoding green fluorescent protein (GFP) fusion proteins

Total RNA was extracted from the leaves of potato (*S. tuberosum* cultivar Pentland Crown) with TRIzol reagent (Invitrogen). cDNA was synthesized from 1 to 2 μg total RNA using *Moloney murine leukemia virus* reverse transcriptase (200 U/μL; Promega, Madison, WI) and random hexamers. The gene encoding the small GTPase rabE1a (PGSC0003DMP400015542) was amplified by PCR from the cDNA using Phusion high-fidelity DNA polymerase (Finnzymes, Espoo, Finland) with primers containing the appropriate restriction sites for cloning. The PCR products were cloned into pRT-GFP vectors as described [21], resulting in plasmids designated as pRT-GFP-rabE1a and pRT-rabE1a-GFP. Subsequently, binary vectors were prepared by transferring the expression cassettes, including the 35S promoter, from the pRT vector backbone to the binary vector pKOH200 using *Hin*dIII.

### Agroinfiltration and fluorescence microscopy

Competent cells of *Agrobacterium tumefaciens* (pGV2260) were transformed with the binary vectors using the freeze-thaw method [73]. Agroinfiltration into leaves of *N. benthamiana* was carried out as previously described [21]. An epifluorescence microscope (Axioimager M2, Carl Zeiss Microscopy GmbH, Jena, Germany) and GFP-compatible and RFP (red fluorescent protein)-compatible filter cubes were used for visualization of expression of GFP and RFP fusion constructs.

## Supplementary information

**Additional file 1: Table S1.** Proteins found in the nucleus of cells of healthy leaves from potato lines v2–108.

**Additional file 2: Table S2.** Proteins detected only in the nucleus of PVA-infected potato leaves.

**Additional file 3: Table S3.** Proteins detected only in the nucleus of healthy potato leaves.

**Additional file 4: Figure S1.** Original images for Fig. 6.

## Data Availability

All data generated in this study are included within the article and its additional files.

## Abbreviations

CP: Coat protein
eIF4E: Eukaryotic initiation factor 4E
ELISA: Enzyme-linked immunosorbent assay
GFP: Green fluorescent protein
GO: Gene ontology
LC-MS/MS: Liquid chromatography–coupled tandem mass spectrometry
NIa: Nuclear inclusion protein a
NIb: Nuclear inclusion protein b
NSAF: Normalized spectral abundance factor
PCR: Polymerase chain reaction
PLGEM: Power law global error model
PVA: Potato virus A
PVY: Potato virus Y
RFP: Red fluorescent protein
TMV: Tobacco mosaic virus
VPg: Viral genome-linked protein
