# Supplementary material for: Nuclear proteome of virus-infected and healthy potato leaves
Source: BMC Plant Biol. 2020 Jul 29;20:355. doi: 10.1186/s12870-020-02561-7 (PMC7392702; doi:10.1186/s12870-020-02561-7)
Supplement: Supplementary file 4 — Additional file 4: Figure S1. Original images for Fig. 6. [file 12870_2020_2561_MOESM4_ESM.docx]

**Figure S1.** Original images for Figure 6. Analysis of nuclear proteins in healthy and PVA-infected potato leaves (line v2-108) used for shotgun proteomics. Histone H3 and luminal-binding protein 2 (BiP2) were detected using specific antibodies. Bottom pane: Coomassie blue (CB) staining of the gel. Lanes: 1, healthy experiment-1; 2, PVA-infected experiment-2; 3, healthy experiment-3; 4, PVA-infected experiment-3; 5, healthy experiment-2; 6, PVA-infected experiment-1; 7, total protein fraction.
